# Low Mercury Concentration Produces Vasoconstriction, Decreases Nitric Oxide Bioavailability and Increases Oxidative Stress in Rat Conductance Artery

**DOI:** 10.1371/journal.pone.0049005

**Published:** 2012-11-07

**Authors:** Núbia Belem Lemos, Jhuli Keli Angeli, Thaís de Oliveira Faria, Eduardo Hertel Ribeiro, Dalton Valentim Vassallo, Alessandra Simão Padilha, Ivanita Stefanon

**Affiliations:** 1 Departamento de Ciências Fisiológicas, Universidade Federal do Espírito Santo, Vitória, Espírito Santo, Brazil; 2 Escola de Ensino Superior da Santa Casa de Misericórdia de Vitória, EMESCAM, Vitória, Espírito Santo, Brazil; Max-Delbrück Center for Molecular Medicine (MDC), Germany

## Abstract

Mercury is an environmental pollutant that reduces nitric oxide (NO) bioavailability and increases oxidative stress, having a close link with cardiovascular diseases, as carotid atherosclerosis, myocardial infarction, coronary heart disease and hypertension. One of the main sites affected by oxidative stress, which develops atherosclerosis, is the aorta. Under acute exposure to low mercury concentrations reactive oxygen species (ROS) production were only reported for resistance vessels but if low concentrations of mercury also affect conductance arteries it is still unclear. We investigated the acute effects of 6 nM HgCl_2_ on endothelial function of aortic rings measuring the reactivity to phenylephrine in rings incubated, or not, with HgCl_2_ for 45 min, the protein expression for cyclooxygenase 2 (COX-2) and the AT1 receptor. HgCl_2_ increased Rmax and pD2 to phenylephrine without changing the vasorelaxation induced by acetylcholine and sodium nitroprusside. Endothelial damage abolished the increased reactivity to phenylephrine. The increase of Rmax and pD2 produced by L-NAME was smaller in the presence of HgCl_2_. Enalapril, losartan, indomethacin, furegrelate, the selective COX-2 inhibitor NS 398, superoxide dismutase and the NADPH oxidase inhibitor apocynin reverted HgCl_2_ effects on the reactivity to phenylephrine, COX-2 protein expression was increased, and AT1 expression reduced. At low concentration, below the reference values, HgCl_2_ increased vasoconstrictor activity by reducing NO bioavailability due to increased ROS production by NADPH oxidase activity. Results suggest that this is due to local release of angiotensin II and prostanoid vasoconstrictors. Results also suggest that acute low concentration mercury exposure, occurring time to time could induce vascular injury due to endothelial oxidative stress and contributing to increase peripheral resistance, being a high risk factor for public health.

## Introduction

Mercury is considered an environmental pollutant of high risk to public health. At present, humans are mostly exposed to mercury by the consumption of mercury-contaminated fish, the administration of thimerosal in vaccines, and the inhalation of mercury vapour during professional exposure [1]–[6]. Mercury compounds are highly volatile and soluble in water and lipids, entering the circulation through the pulmonary alveolus and by intestinal absorption, and crossing the blood-brain barrier. Once absorbed mercury produces adverse effects as kidney damage, acrodynia, gastroenteritis, pneumonia and pulmonary fibrosis, reduction of reproductive function and infertility, and affects the cardiovascular system, among others. [1], [4], [6]–[9].

Numerous studies have shown that mercury might induce oxidative stress with subsequent damage to several organs or systems [10]–[14] and also to reduce nitric oxide (NO) production and to suppress the inducible NO synthase gene expression [15]–[16]. Indeed, there is a close link between mercury and cardiovascular diseases, such as carotid atherosclerosis, myocardial infarction, coronary heart disease and hypertension [5]–[6], [10]. Mercury exists in several forms: inorganic mercury as metallic mercury and mercury vapor (Hg^0^) and mercurous mercury (Hg^+^) or mercuric mercury (Hg^++^) salts, and organic mercury, also called organometallic. The biological behavior, pharmacokinetics, and clinical significance of the various forms of mercury vary according to its chemical structure [17]. Once in the bloodstream, mercury undergoes catalase and peroxidase-mediated oxidation in red blood cells and tissues and is transformed into inorganic mercuric mercury (Hg^++^) and mercurous mercury (Hg^+^) [10], [18]. Methylmercury is by far the most common form of organic Hg to which humans and animals are exposed and it is predominantly formed by methylation of inorganic mercuric ions by microorganisms present in soil and water [19]–[21].

Oxidative stress is known as an efficient mechanism to produce oxidized low-density lipoprotein and consequently atherosclerosis [22]. Advanced glycation end products are generated and subsequent participation of inflammatory cells maintains vascular injury [23]. Mercury effects after chronic exposure generating oxidative stress at endothelial level are already reported for both conductance and resistance vessels [13], [24]–[25]. However, under acute exposure to low mercury concentrations (6 nM) reactive oxygen species (ROS) production were only reported for resistance vessels [26].

One important site affected by oxidative stress, which develops atherosclerosis, is the aorta. However, if short periods of exposure and if low concentrations of mercury also affect conductance arteries it is still unclear [13], [26]. The fact that the endothelium is affected by low concentrations of heavy metals below the reference values highlights the importance and the need to better understand the mechanisms by which these metals promote development of cardiovascular diseases [27]–[28]. Therefore, the current study aimed to explore the acute effect of low-concentration mercury exposure on endothelial function in the isolated aortic rings of rats and the possible role of oxidative stress and prostanoids generation. Our findings suggest that just a brief exposure of arteries to low mercury concentration, increases oxidative stress and render them hypercontractile secondary to an immediate loss of NO bioavailability.

**Figure 1:**
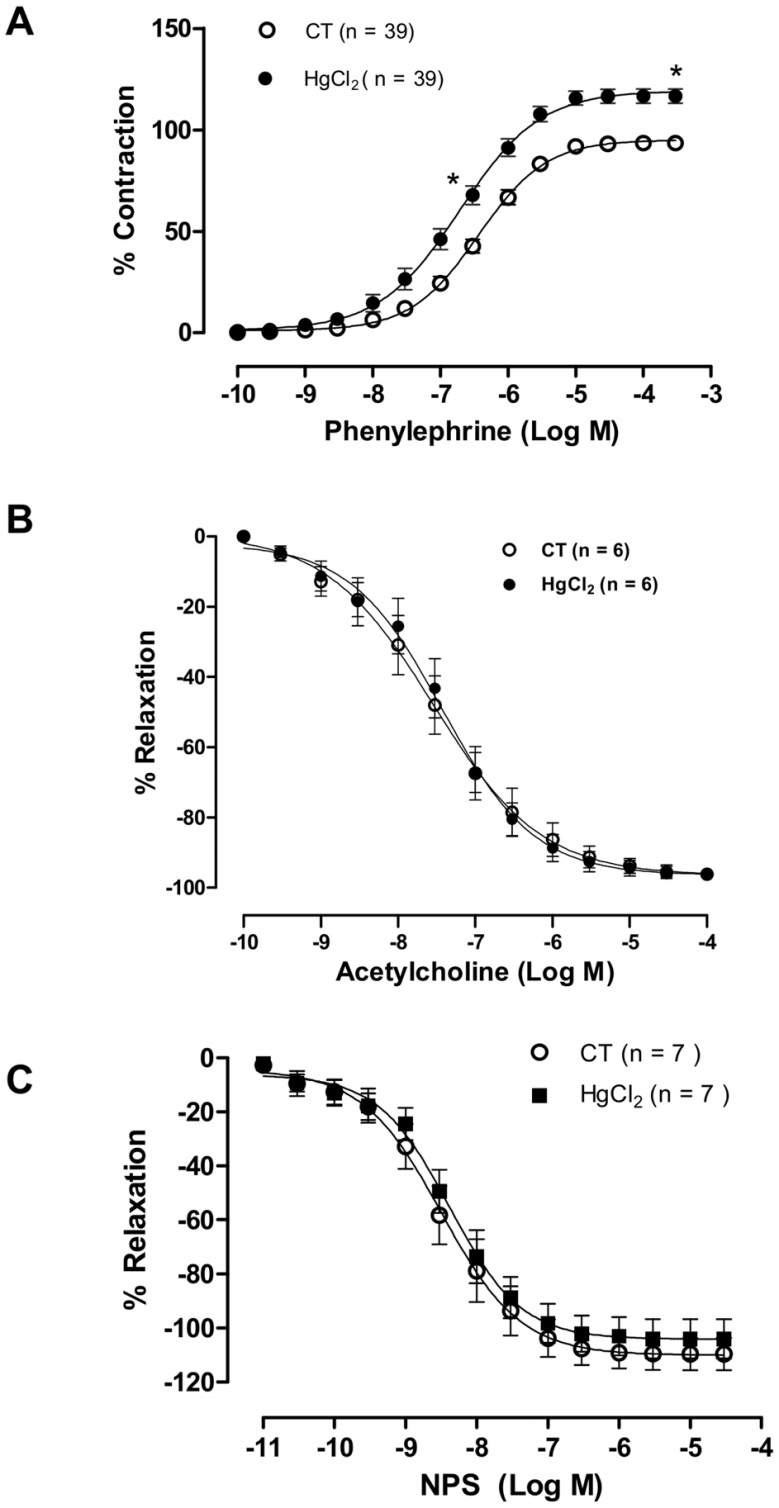
The effect of acute incubation with mercury chloride (HgCl2) on the concentration-response curves for phenylephrine (A), acetylcholine (B), and sodium nitroprusside (NPS) (C) in aortic rings in control (CT) conditions or HgCl2. Results (mean±SEM) are expressed as a percentage of the response to 75 mmol/l KCl and percentage of the relaxation to phenylephrine respectively. *P<0.05 by ANOVA. Number of animals used in parentheses.

**Table 1:** Parameters of maximal response (Emax, mmHg) and sensitivity (pD2) of dose-response curves to phenyleprine in the aorta, before (E+) and after (E-) endothelial damage and after NG-nitro-L-arginine methyl ester (L-NAME, 100 µM) incubation, in the presence (HgCl2 E+) and absence of mercury chloride (E- HgCl2).

	n	Emax (%)	pD_2_
CT E+	39	93.5±2.52	−6.47±0.08
HgCl_2_ E+	39	117±3.45 *	−6.77±0.10*
CT E-	07	128±3.08	−8.06±0.04
HgCl_2_ E-	08	129±3.37	−8.34±0.24
CT E+	06	93±4	−6.4±0.09
L-NAME CT	06	183±15.3 *	−7±0.09 *
HgCl_2_ E+	05	113±4.78	−6.6±0.14
L-NAME + HgCl_2_	05	168±8.94 *	−7±0.05 *

**Figure 2:**
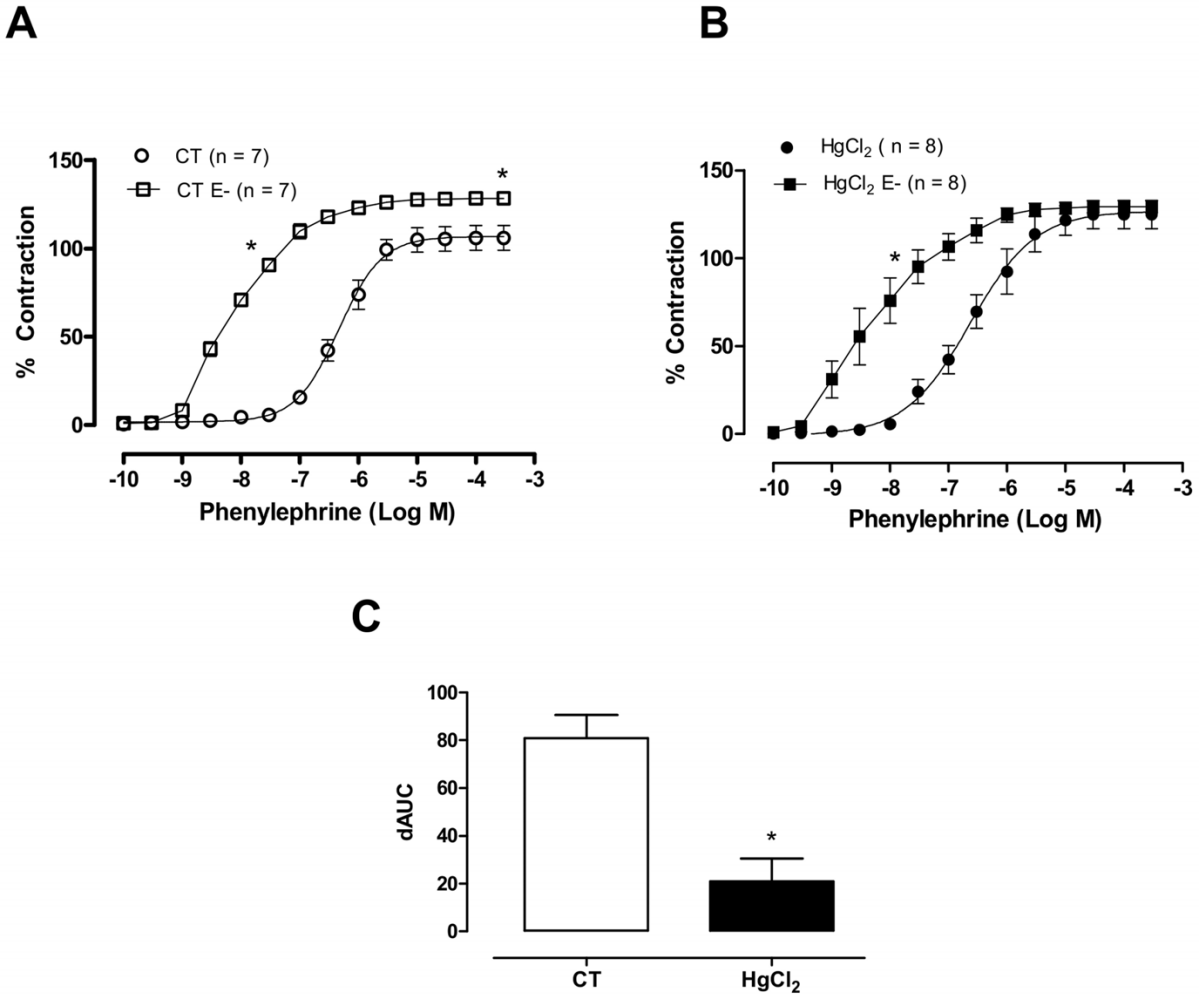
The effect of endothelium removal (E-) on the concentration-response curves for phenylephrine in aortic rings in control (CT) conditions or HgCl2 (A,B). Results (mean±SEM) are expressed as a percentage of the response to 75 mmol/l KCl. *P<0.05 by ANOVA. Number of animals used in parentheses. (C) Differences in area under the concentration-response curve (dAUC) in the presence and absence of the endothelium of aortic rings in control (CT) conditions or HgCl_2_. *P<0.05 by Student’s *t*-test.

**Figure 3:**
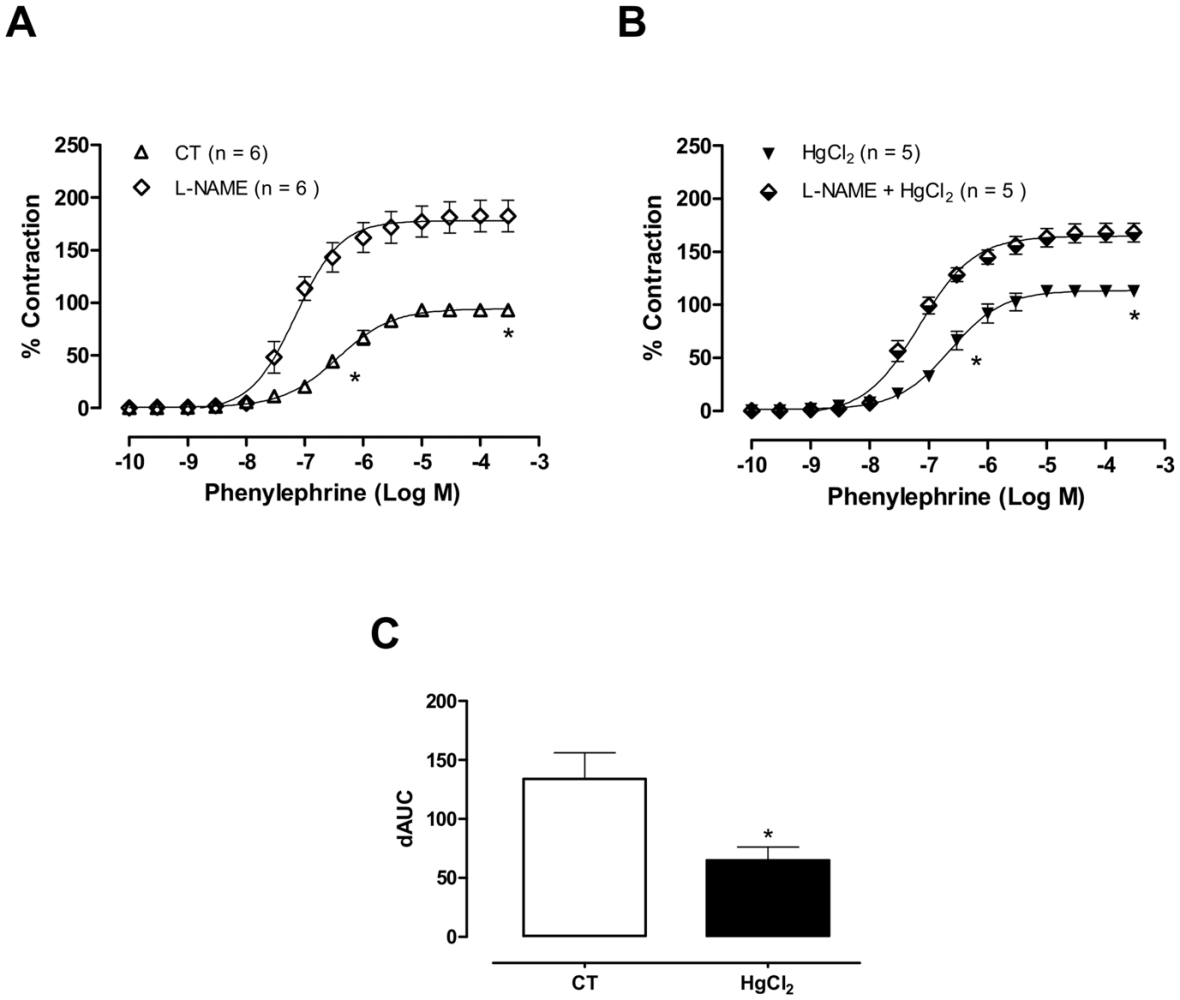
The effect of L-NAME (100 µM) on the concentration-response curves for phenylephrine in aortic rings in control (CT) conditions or HgCl2 (A,B). Results (mean±SEM) are expressed as a percentage of the response to 75 mmol/l KCl. *P<0.05 by ANOVA. Number of animals used in parentheses. (C) Differences in the area under the concentration-response curve (dAUC) for aortic rings incubated in the presence and absence of L-NAME in controls (CT) or HgCl_2_. *P<0.05 by Student’s *t*-test.

Results are expressed as means ± SEM of the no. of animals shown in Figs. 1–3. P<0.05 vs. CT (E+) (*).

## Materials and Methods

### Animals

Studies were performed on male Wistar rats (250–300 g). All experiments were conducted in compliance with the guidelines for biomedical research as stated by the Brazilian Societies of Experimental Biology and approved by a local ethic committee (004/2007 CEUA-EMESCAM). All rats had free access to water and were fed rat chow *ad libitum*.

### Isolated Rat Aorta Preparation

Rats were anesthetised with sodium thiopental (50 mg/kg, i.p.) and euthanized by exsanguination. Thoracic aortas were carefully dissected out and cleaned of fat and connective tissue. For reactivity experiments, the aortas were divided into cylindrical segments of 4 mm in length (5 to 6 each aortic rings). For the analysis of cyclooxygenase 2 (COX2) isoform and AT1 protein expression, after the experiments, arteries were rapidly frozen in liquid nitrogen and kept at −70°C until the day of analysis.

#### Vascular reactivity measurements

Segments of thoracic aorta were mounted in an isolated tissue chamber containing Krebs–Henseleit solution (in mM: NaCl 118; KCl 4.7; NaHCO_3_ 23; CaCl_2_ 2.5; KH_2_PO_4_ 1.2; MgSO_4_ 1.2; glucose 11 and EDTA 0.01), gassed with 95% O_2_ and 5% CO_2_ and maintained at a resting tension of 1 g at 37°C. Isometric tension was recorded using an isometric force transducer (TSD125C, CA, U.S.A) connected to an acquisition system (MP100 Biopac Systems, Inc., Santa Barbara, CA, U.S.A.).

After a 45-min equilibration period, all aortic rings were initially exposed twice to 75 mM KCl, first to check their functional integrity and again to assess the maximal tension that developed. Afterwards, endothelial integrity was tested with acetylcholine (10 µM) in segments that were previously contracted with phenylephrine (1 µM). Relaxation equal to or greater than 90% was considered demonstrative of the functional integrity of the endothelium. After a washout period (30 min), the aortic rings were incubated with HgCl_2_ (6 nM) or not (control) for 45 min. Then, increasing concentrations of phenylephrine (0.1 nM–30 mM) were applied, a concentration–response curve to this contractile agonist was obtained and tension was measured once a plateau was attained.

**Figure 4:**
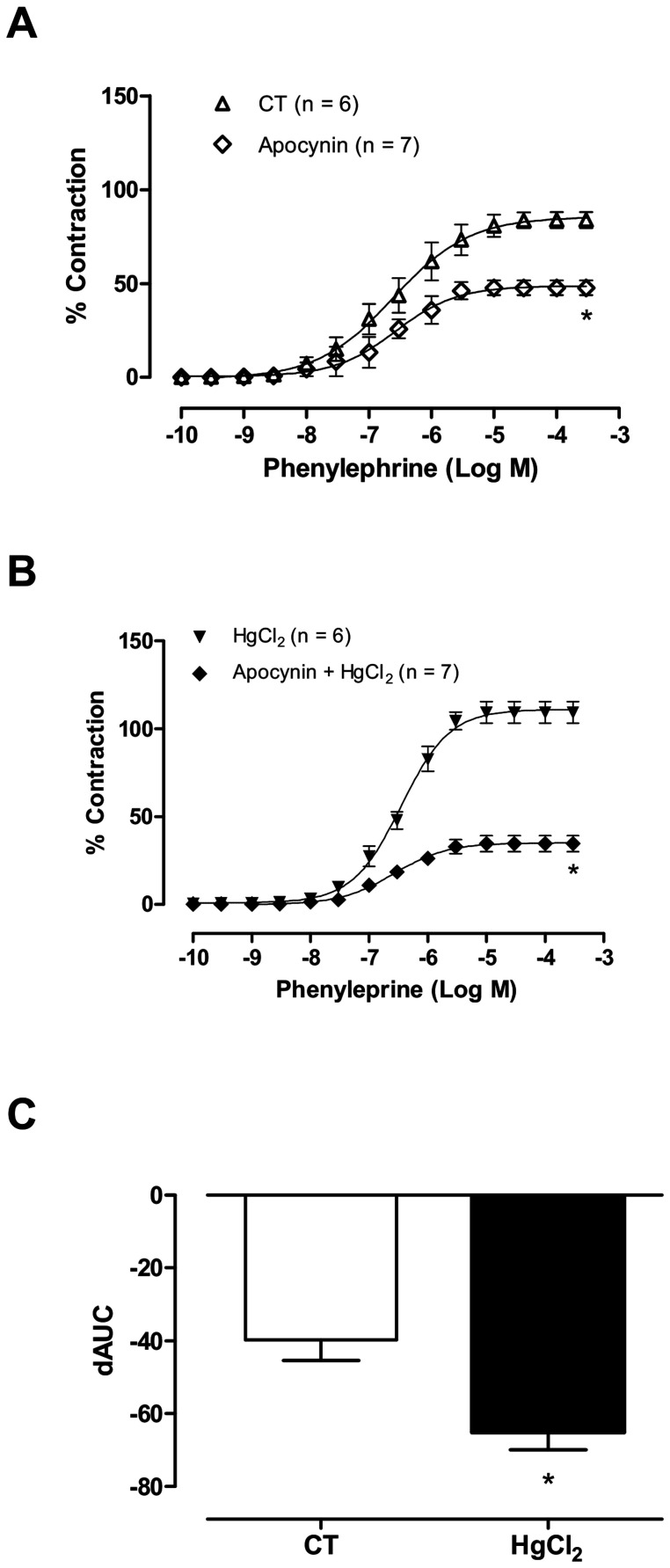
The effect of apocynin (0.3 mM) on the concentration-response curves for phenylephrine in aortic rings in control (CT) conditions or HgCl2 (A,B). Results (mean±SEM) are expressed as a percentage of the response to 75 mmol/l KCl. *P<0.05 by ANOVA. Number of animals used in parentheses. (C) Differences in the area under the concentration-response curve (dAUC) in aortic rings cultured the presence and absence apocynin (0.3 mM), under control (CT) conditions or HgCl_2_. *P<0.05 by Student’s *t*-test.

**Figure 5:**
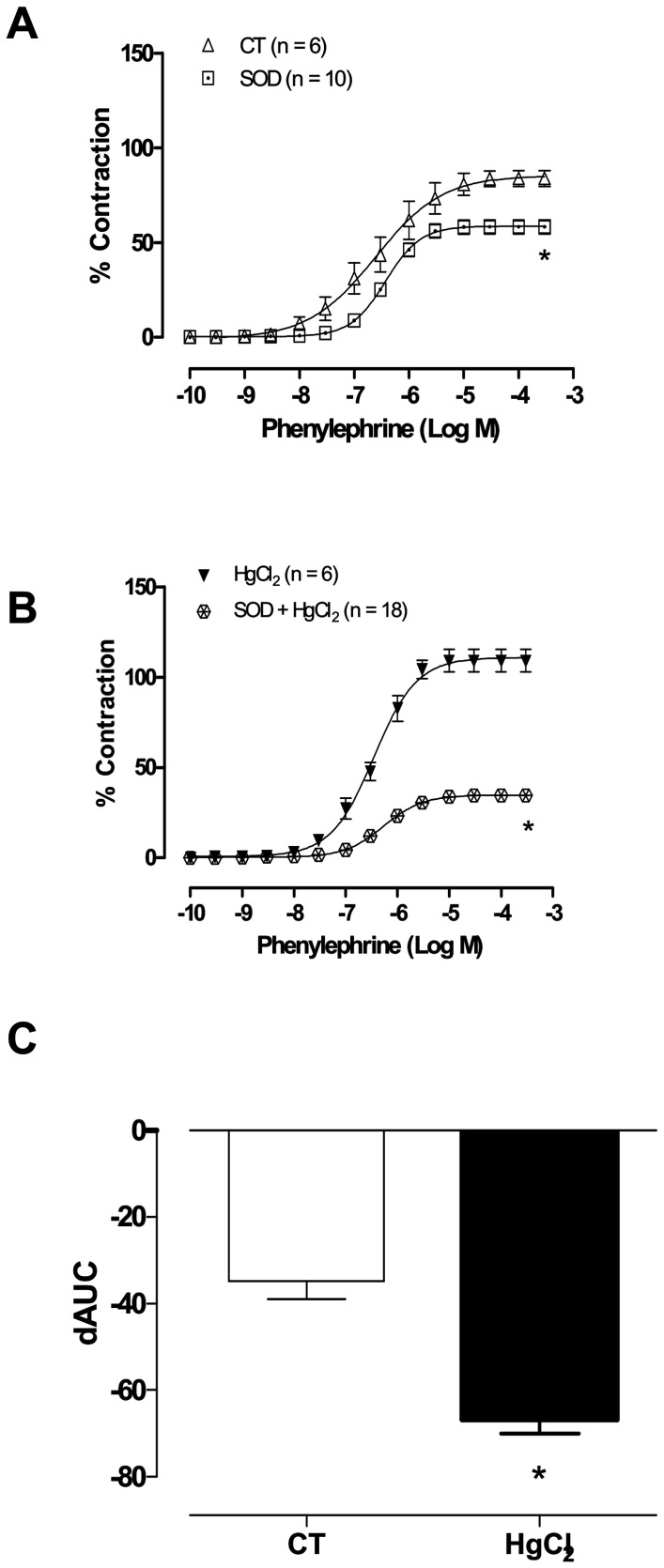
The effect of superoxide dismutase (SOD, 150 U ml^−**1**^**) on the concentration-response curves for phenylephrine in aortic rings in control (CT) conditions or HgCl2 (A,B).** Results (mean±SEM) are expressed as a percentage of the response to 75 mmol/l KCl. *P<0.05 by ANOVA. Number of animals used in parentheses. (C) Differences in the area under the concentration-response curve (dAUC) in aortic rings cultured in the presence and absence of superoxide dismutase (SOD, 150 U ml^−1^) under control (CT) conditions or HgCl_2_. *P<0.05 by Student’s *t*-test.

For this study we selected the concentration of 6 nM HgCl_2_, as a single concentration to be used in these experiments based on previous findings in our laboratory [26]. However, previous reports suggested that exposure to mercury vapor [2], due to the release of mercury from or during removal of amalgam fillings [29], increase blood and plasma concentrations (4 to 5 nM). However, toxicological consequences were still a matter of debate. We previously investigated the effects of continuous perfusion of 6 nM HgCl_2_ on the vascular reactivity of the perfused tail artery of the rat [26], which are not typical conductance arteries. We also investigated a chronic treatment focused on resistance, conductance and coronary arteries [13], [24]–[25]. We then performed experiments to know if low mercury concentrations, without time to concentrate inside cells, could affect conductance arteries, as occurred for the tail artery. Knowing that oxidative stress and reduction of nitric oxide bioavailability, which are involved in aortic atherosclerosis development, these experiments were performed. If results did appear the repetitive exposure would gain importance.

**Figure 6:**
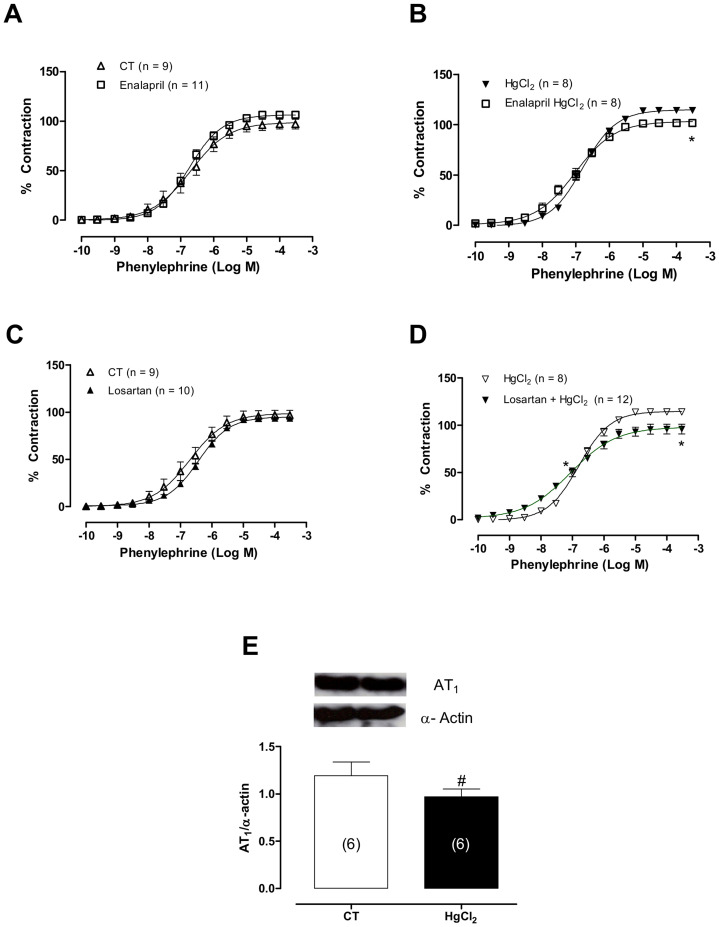
(A,B) The effects of enalapril (10 µM) and (C,D) losartan (10 µM) on the concentration-response curves for phenylephrine and (E) densitometric analysis of the western blot for AT_1_ in aortic rings in control (CT) conditions or HgCl2. Results (mean±SEM) are expressed as a percentage of the response to 75 mmol/l KCl. *P<0.05 by ANOVA. (E) Densitometric analysis of the western blot for angiotensin receptor 1 (AT1) protein expression in aortic rings cultured in the absence (CT) of HgCl_2_ and after acute incubation with HgCl_2_. *P<0.05 by Student’s *t*-test. Number of animals used in parentheses. Representative blots are also shown.

**Figure 7:**
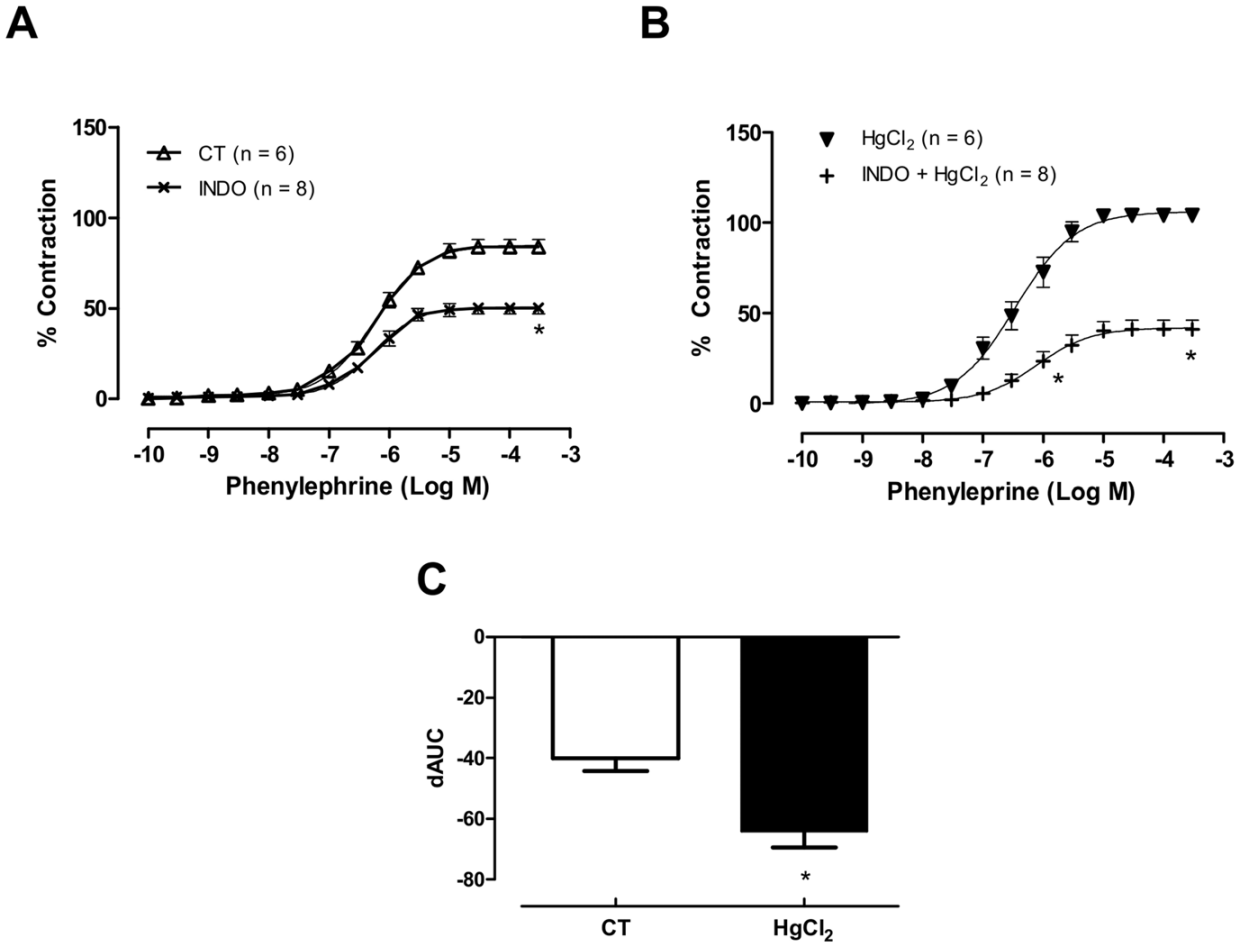
(A, B) The effect of indomethacin (Indo, 10 µM), on the concentration-response curves for phenylephrine in aortic rings in control (CT) conditions or HgCl2. Results (mean±SEM) are expressed as a percentage of the response to 75 mmol/l KCl. *P<0.05 by ANOVA. Number of animals used in parentheses. (C) Differences in the area under the concentration-response curve (dAUC) in aortic rings cultured in the presence of indomethacin (10 µM) under control (CT) conditions and after acute incubation with mercury HgCl_2_. *P<0.05 by Student’s *t*-test.

**Figure 8:**
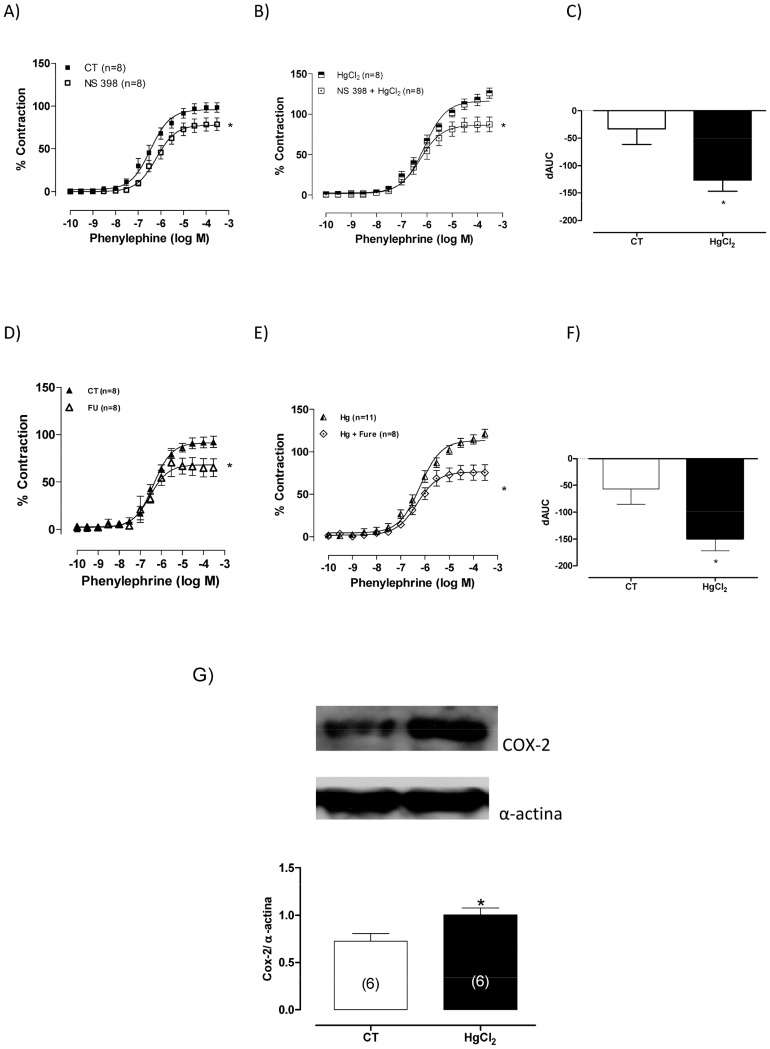
(A,B) The effect of NS 398 (10^−6^ M) and (D,E) furegrelate (FU, 10^−3^ M) on the concentration-response curves for phenylephrine in aortic rings in control (CT) conditions or HgCl2. Results (mean±SEM) are expressed as a percentage of the response to 75 mmol/l KCl. *P<0.05 by ANOVA. Number of animals used in parentheses. (C, F) Differences in the area under the concentration-response curve (dAUC) in aortic rings cultured in the presence of NS 398 (10^−6^ M) and furegrelate (FU, 10^−3^ M) under control (CT) conditions and after acute incubation with mercury HgCl2. *P<0.05 by Student’s *t*-test. Densitometric analysis of the western blot for cycloxxigenase-2 (COX-2) protein expression in aortic rings in control (CT) conditions or HgCl2. *P<0.05 by Student’s *t*-test. Number of animals used in parentheses. Representative blots are also shown.

The influence of the endothelium on the response to phenylephrine in the absence or presence of HgCl_2_ (6 nM) was investigated after its mechanical removal by rubbing the lumen with a needle. The absence of endothelium was confirmed by the inability of 10 µM acetylcholine to produce relaxation. The role of endothelial derived vasoactive factors on the phenylephrine-elicited contractile response was investigated. The effects of the following drugs were evaluated: 1) a nonspecific NOS inhibitor N-nitro-L-arginine methyl ester (L-NAME, 100 µM), 2) an enzyme scavenger of ROS (superoxide dismutase, 150 U ml^−1^), 3) an NADPH oxidase inhibitor (apocynin, 0.3 mM), 4) an angiotensin converting enzyme inhibitor (enalapril, 10 µM), 5) an angiotensin II type 1 receptor antagonist, (losartan, 10 µM), 6) a non-selective cyclooxygenase (COX) inhibitor (indomethacin, 10 µM), 7) a COX-2 inhibitor (NS 398, 1 µM), and 8) a thromboxane A(2) (TXA_2_) synthase inhibitor (furegrelate, 1 mM). These drugs were added 45 min before the generation of the concentration–response curves to phenylephrine, in the absence or presence of HgCl_2_ (6 nM).

In another set of experiments, after the 45-min equilibration period, aortic rings were contracted with phenylephrine (1 µM) until reaching a plateau (about 15 min), and the concentration-response curves to acetylcholine (0.1 nM–30 mM) or sodium nitroprusside (0.01 nM–30 µM) were obtained before and after incubation with HgCl_2_ (6 nM), for 45 min.

### Biochemical Studies

#### Western blot analysis of COX-2 and AT_1_ expression

COX-2 and AT1 protein expression were determined in homogenates from the aortic segments used for the reactivity experiments (approximately 6 h after extraction from the animal) that had been pre-incubated with or without HgCl_2_ (for 45 min) as previously described [30].

Proteins from homogenised arteries (90 µg of protein) were separated by 10% SDS-PAGE. Proteins were transferred to nitrocellulose membranes that were incubated with either mouse monoclonal antibodies for COX-2 (1∶200; Cayman Chemical; Ann Arbor, MI, USA) and AT_1_ (1∶500, Santa Cruz, Biotechnology, Santa Cruz, CA). After washing, the membranes were incubated with anti-mouse or anti-rabbit (1∶5000, StressGen, Victoria, Canada) immunoglobulin antibody conjugated to horseradish peroxidase. After thorough washing, immunocomplexes were detected using an enhanced horseradish peroxidase/luminal chemiluminescence system (ECL Plus, Amersham International, Little Chalfont, UK) and film (Hyperfilm ECL International). Signals on the immunoblot were quantified with the National Institutes of Health Image V1.56 computer program. The same membrane was used to determine α-actin expression using a mouse monoclonal antibody (1∶5000, Sigma, USA).

#### Nitric oxide release

Nitric oxide release was measured in thoracic aorta segments. Thoracic aorta segments were dissected and equilibrated for 30 min in HEPES buffer (in mmol·L^−1^: 119 NaCl, 20 HEPES, 1.2 CaCl_2_, 4.6 KCl, 1 MgSO_4_, 0.4 KH_2_PO_4_, 5 NaHCO_3_, 5.5 glucose, 0.15 NaH_2_PO_4_; pH 7.4) at 37°C. Arteries were then incubated with the fluorescent probe 4,5-diaminofluorescein (2 mmol·L^−1^) for 30 min, and the medium was collected to measure basal NO release. NO release was evaluated by measuring the release of NO after incubation with or without HgCl_2_ for 45 minutes at 37°C. The fluorescence of the medium was measured at room temperature using a spectrofluorometer (LS50 Perkin Elmer Instruments, FL WINLAB Software) with the excitation wavelength set at 492 nm and the emission wavelength set at 515 nm. Blank measurement samples were similarly collected but without arteries to subtract background emission. The amount of NO released was expressed as arbitrary units·mg^−1^ tissue.

### Drugs and Reagents

HgCl_2_, l-phenylephrine hydrochloride, L-NAME, enalaprilate, indomethacin, acetylcholine chloride, sodium thiopental, losartan, apocynin, sodium nitropusside, SOD, were purchased from Sigma-Aldrich (St Louis, MO, USA); furegrelate and NS 398 were purchased from Cayman Chemical (Ann Arbor, MI) and 4,5-diaminofluorescein (Sigma Aldrich). Salts and reagents when not specified were of analytical grade obtained from Sigma (St Louis, MO, USA) and Merk (Darmstadt, Germany).

### Data Analysis

Vasoconstrictor responses induced by phenylephrine were normalised to the contraction induced by 75 mM KCl and expressed as a percentage of this contraction. Vasodilator responses are expressed as the percentage of the previous contraction. For each concentration-response curve, the maximum effect (Rmax) and the concentration of agonist that produced one-half of Rmax (EC50) were calculated using nonlinear regression analysis (GraphPad Prism Software, San Diego, CA). The sensitivity of the agonists was expressed as pD2 (-log EC50). To compare the effects of L-NAME or endothelium denudation on the contractile response to phenylephrine, the results were expressed as differences in the area under the concentration-response curves (dAUC) for phenylephrine in control and experimental situations. AUCs were calculated from the individual curve plots (GraphPad Prism Software), and differences are expressed as the percentage of the AUC for the corresponding control situation. These values indicate whether the magnitude of the effect of each treatment is different after incubation with HgCl_2_. For protein expression, data are expressed as the ratio between signals on the immunoblot corresponding to the studied protein and α-actin.

Results are expressed as the means ± SEM of the number of rats studied; differences were analysed using Student’s *t*-test or one-way ANOVA followed by a Bonferroni test. *P*<0.05 was considered significant.

## Results

### Effect of Mercury Incubation on Vasoconstrictor and Vasodilator Responses

Acute mercury incubation increased the maximal response (Rmax) and sensitivity (pD2) to phenylephrine in aortic rings when compared to controls, but it did not modify the endothelium-dependent and -independent relaxation induced by acetylcholine and sodium nitroprusside, respectively (Figure 1, Table 1).

Endothelium removal (Fig. 2A and 2B; Table 1) and incubation with the NOS inhibitor L-NAME (100 µM; Figure 3; Table 1) left-shifted the concentration-response curves to phenylephrine in aortic segments from both groups. These effects were smaller in mercury-treated rings, as shown by the dAUCs (Figure 2C and 3C). These results indicate that mercury incubation decreased NO bioavailability.

### The Role of Free Radicals in the Effects of HgCl_2_ on the Phenylephrine Response

To determine if the endothelial damage observed in aortic rings after mercury incubation was related to changes in O_2_^_^ production, the effects of the NADPH oxidase inhibitor apocynin and superoxide anion scavenger SOD on vasoactive responses were investigated. Apocynin (0.3 mM; Figure 4A, 4B) and SOD (150 U/ml; Figure 5A and 5B) reduced both vasoconstrictor responses to phenylephrine in control aortic segments and those that had been incubated with mercury. However, the magnitude of these effects was greater after incubation with mercury, as shown by the dAUCs (Ct −39.8±5.7 vs HgCl_2_ −65.1±4.8 Figure 4C and Ct −39.4±4.1 vs HgCl_2_ −66.8±3.2 Figure 5C).

### The Role of the Renin-Angiotensin System in the Effects of HgCl_2_ on the Phenylephrine Response

To investigate whether the local renin-angiotensin-system is involved in the mercury-induced changes in vascular reactivity to phenylephrine, AT_1_ receptors and angiotensin converting enzyme activity were blocked with losartan (10 µM) or enalapril (10 µM), respectively. As shown in the Figure 6 (A, B, C and D), both drugs partially reduced the effects of mercury on the concentration-response curve to phenylephrine (pD2: Ct, 6.73±0.06; HgCl_2_, 7.03±0.01; Enalapril, 6.76±0,18; Enalapril + HgCl_2_, 6,07±0,03; Losartan, 6.8±0,3; Losartan + HgCl_2_, 7.03±0.02 Emax: Ct, 93.5±2.52; HgCl_2_, 115.04±3.12; Losartan, 97.04±4.09; Enalapril, 106.28±3.02; Losartan+ HgCl_2_ 95.81±3.02; Enalapril+ HgCl_2_ 101.6±2.91). Corroborating these data, the protein expression of AT_1_ was reduced after acute exposure to mercury (Figure 6E).

### The Role of the Cyclooxygenase Pathway in the Effects of HgCl_2_ on the Phenylephrine Response

To investigate the putative role of prostanoids on the enhanced response to phenylephrine produced by the incubation of the aortic rings with HgCl_2_, indomethacin (10 µM) was used. It reduced the phenylephrine response in aortic rings from both control and Hg treated groups with indomethacin, and this effect was greater after incubation with mercury (Figure 7A and 7B); dAUC indomethacin (Figure 7C), Ct −40.01±4.22 vs HgCl_2_ −64.10±5.36. To clarify this issue, we investigated whether cyclooxygenase II or thromboxane A2 contributed to the effects of mercury. Aortic rings were incubated with NS 398 (1 µM; Figure 8A and 8B), an inhibitor of COX2, and furegrelate (10 µM; Figure 8D and 8E), an inhibitor of TXA2. As shown in Figure 8C and 8F, both drugs reduced the effects of mercury without altering the vascular reactivity to phenylephrine in control rings (dAUC: NS398, Ct −32.7±29.02 vs HgCl_2_ −126.4±20.5) and (dAUC: furegrelate, Ct −52.65±28.55 vs HgCl_2_ −149.26±22.83). In addition, COX-2 protein expression also increased in aortic rings after acute exposure to mercury (Figure 8G).

## Discussion and Conclusions

The present study demonstrated that even at a low concentration (6 nM), acute mercury administration alters the endothelial function of conductance vessels, as previously reported for resistance vessels [13], [26]. Reduction of NO after exposure to mercury promoted decreased the NO endothelial modulation due to increased O_2_^−^ production increasing the vasoconstrictor response to phenylephrine. Administration of SOD and the NADPH oxidase inhibitor, apocynin, reduced this increased vasoconstrictor response. Results from pharmacological interventions with losartan, enalapril, indomethacin, NS 398 and furegrelate suggest that the renin-angiotensin system and COX-2 might be involved in the vascular effects of mercury. In addition, our results show an increase in COX-2 protein expression and a decrease in AT1 protein expression.

The fact that the endothelium is affected by low concentrations of heavy metals below the reference values highlights the importance and the need to better understand the mechanisms by which these metals promote development of cardiovascular diseases [8], [27]. Mercury induces toxicological consequences as a result of exposure to mercury vapour [2], the release of mercury from or during removal of amalgam fillings [29] and the ingestion of contaminated fish [4]. Exposure to mercury vapour and removal of amalgam fillings increase blood and plasma concentrations of the metal (4 to 5 nM), but the toxicological consequences are still a matter of debate. In a recent biomonitoring study in New York City adults, the blood mercury concentration was 2.73 ng/mL, whereas in regular fish consumers it reached 5.65 ng/mL [3]. In the present study, we used a low concentration of HgCl_2_ (6 nM = 1.6 ng/mL), which is similar to the levels reached in exposed humans.

In some pathological conditions, such as atherosclerosis and hypertension, a reduction in NO bioavailability is reported to contribute to the appearance of cardiovascular disease [25]. Thus, a decrease in NO production could contribute for the development of cardiovascular disease. The main factor that contributes to the reduction of NO bioavailability is the increase of ROS. The superoxide anion interacts with NO and forms peroxynitrite, thereby decreasing the NO bioavailability for smooth muscle relaxation [31]. Thus, the reduced NO bioavailability induced by mercury could be related to increased levels of ROS. In fact, previous studies have associated mercury exposure with increased oxidative stress [5], [11], .

NO reduction is reported to increase vascular reactivity to vasoconstrictors [34]. We observed that acute administration of HgCl_2_ promoted an increase in reactivity to phenylephrine in aortic rings. The comparison of dAUC changes of aortic rings with and without endothelium, suggested that the magnitude of the response to phenylephrine was lower after incubation with mercury. This result suggests that the ability of the endothelium to negatively modulate the contractile response induced by phenylephrine seems to be impaired in isolated aortic rings incubated with HgCl_2_.

We then investigated if the reduction of endothelial modulation after incubation with mercury in aortic rings could be a consequence of reduced NO bioavailability as previously reported in the tail vascular bed and in mesenteric and aortic arteries [13], [26], [34]. In this study we also found reduced levels of NO after exposure to HgCl_2_. Grotto and colleagues [35] also reported a depletion of NO in rats sub-chronically exposed to MeHg at a low level.

However, an underlying mechanism associated to those previous findings is the oxidative stress generation. Our results showed that treatment with SOD or apocynin were able to reverse the effects of mercury on the vascular reactivity to phenylephrine in aortic rings, suggesting that acute exposure to mercury is accompanied by an increased production of ROS. Therefore, we suggest that increased vascular reactivity to phenylephrine induced by mercury could have been caused by an increased release of ROS with the resulting reduced bioavailability of NO.

We previously reported that treatment with small doses of mercury increases the release of COX-2 derived vasoconstrictor prostanoids and its participation in vasoconstrictor responses [20]. The increased activation of the renin-angiotensin system after mercury treatment would be associated with this increased COX-2 activity. These mechanisms would contribute to explain the endothelial dysfunction and the increased vasoconstrictor responses induced by mercury exposure [24]. A previous report has shown that an increase of the activity of the local renin-angiotensin system and production of prostanoid vasoconstrictors could increase ROS in the aorta of normotensive (WKY) and spontaneously hypertensive rats (SHR) [30]. Therefore, we investigated whether the vascular actions induced by mercury could involve the renin-angiotensin system and the cyclooxygenase pathway. Our findings indicate that HgCl_2_ is able to increase the release of angiotensin II, which in turn could promote down regulation of AT_1_ protein expression. In addition, angiotensin II can regulate COX-2 protein expression, the production of prostanoids [30], [33], [36], the ROS production [32] and NADPH oxidase activity [33], [37]. Thus, increased local release of angiotensin II induced by mercury could cause the increased activity of COX-2 and NADPH oxidase and this could increase the release of ROS, as observed in this study.

In addition, we observed that indomethacin, NS 398 and furegrelate were able to reduce the effect of mercury on the contractile response to phenylephrine. These results suggest that vasoconstrictor prostanoids, specifically thromboxane A2, participate in the increased reactivity to phenylephrine induced by mercury. Corroborating these results, we also observed that acute exposure to HgCl_2_ increased the protein expression of COX-2. The increased protein expression of COX-2 may result in an increased release of vasoconstrictor prostanoids and in an overproduction of ROS [32], [38]. Taken together, these factors might contribute to inflammation, vascular injury and atherosclerosis development, rendering the vascular hyperreactivity to phenylephrine induced by mercury.

Although our results suggest a reduction of NO bioavailability induced by mercury, relaxations induced by acetylcholine and sodium nitroprusside were not altered after incubation of aortic rings with this metal. These results indicate that the release of NO stimulated by acetylcholine was unaffected by incubation with mercury, suggesting that mercury does not seem to acutely modify the activity of NOS or signalling pathways involving guanylate cyclase. In contrast, a previous study has reported alterations in NOS activity due to sub-chronic exposure to methyl mercury [35]. Kishimoto and colleagues [39] also reported inhibition of NOS in cultured human umbilical vascular endothelial cells as a result of mercury toxicity. However, these studies were performed after sub-chronic exposure to mercury [35] or exposure to high concentrations of this metal [39].

However, our findings do not allow us to define which mechanism is the main one induced by mercury to increase oxidative stress, at least under the present experimental conditions. NO bioavailability reduction caused by ROS or increased endothelial production of vasoconstrictors, angiotensin II and tromboxane A2, altogether increase vascular reactivity.

In summary, these results show that in vitro exposure to low concentrations of HgCl_2_ (6 nM) increased vascular reactivity to phenylephrine, possibly as a result of reduced NO bioavailability due to increased release of ROS. The increased release of ROS derived from NADPH oxidase might be due to the increased local release of angiotensin II and the release of prostanoid vasoconstrictors derived from the COX-2 pathway. Acute mercury exposure, occurring time to time could induce inflammation, vascular injury and atherosclerosis due to endothelial oxidative stress and contributing to increase peripheral resistance. Results also suggest that time to time acute exposure even at low mercury concentration might be a high risk factor for public health.
